# Genetic diversity patterns of lionfish in the Southwestern Atlantic Ocean reveal a rapidly expanding stepping-stone bioinvasion process

**DOI:** 10.1038/s41598-023-40407-y

**Published:** 2023-08-18

**Authors:** Rodrigo Maggioni, Rafael S. Rocha, Jhonatas T. Viana, Tommaso Giarrizzo, Emanuelle F. Rabelo, Carlos E. L. Ferreira, Claudio L. S. Sampaio, Pedro H. C. Pereira, Luiz A. Rocha, Tallita C. L. Tavares, Marcelo O. Soares

**Affiliations:** 1 Instituto de Ciências do Mar (LABOMAR), Universidade Federal do Ceará (UFC), Avenida da Abolição, Fortaleza, 3207 Brazil; 2 Núcleo de Ecologia Aquática e Pesca da Amazônia (NEAP), Universidade Federal do Pará (UFPA), Belém, PA Brazil; 3 Universidade Federal Rural do Semiárido (UFERSA), Mossoró, Brazil; 4 Laboratório de Ecologia e Conservação de Ambientes Recifais (LECAR), Departamento de Biologia Marinha, Universidade Federal Fluminense (UFF), Niterói, RJ Brazil; 5 Unidade Penedo, Universidade Federal de Alagoas (UFAL), Alagoas, Brazil; 6 Projeto Conservação Recifal (PCR), Recife, Brazil; 7 California Academy of Sciences, San Francisco, USA; 8 Reef Systems Group, Leibniz Center for Tropical Marine Research (ZMT), Bremen, Germany; 9 Center for Marine and Environmental Studies (CMES), University of the Virgin Islands (UVI), St. Thomas, US Virgin Islands

**Keywords:** Haplotypes, Sequencing, Biogeography, Molecular ecology, Population genetics, Genetic variation

## Abstract

In 2020, multiple lionfish (*Pterois* spp.) records along the equatorial Southwestern (SW) Atlantic revealed a new expansion of these potentially damaging invasive populations, which could impact over 3500 km of Brazilian coastline over the next few years, as well as unique ecosystems and marine protected areas in its path. To assess the taxonomic status, invasion route, and correlation with other centres of distribution, we investigated the genetic diversity patterns of lionfish caught in 2022 at the Amazonia, Northeastern Brazil, and Fernando de Noronha and Rocas Atoll ecoregions, using two molecular markers, the mitochondrial COI and the nuclear S7 RP1. The data indicate that all studied lionfish belong to what is generally accepted as *P. volitans*, and share the same genetic signature as lionfish present in the Caribbean Sea. The shared haplotypes and alleles indicate that the SW Atlantic invasion derives from an active movement of adult individuals from the Caribbean Sea into the Brazilian coast. The Amazon mesophotic reefs likely served as a stepping-stone to overcome the biogeographical barrier represented by the Amazon-Orinoco River plume. New alleles found for S7 RP1 suggest the onset of local genetic diversification, heightening the environmental risks as this bioinvasion heads towards other South Atlantic ecoregions.

## Introduction

Biological invasions have become one of the leading drivers of biodiversity loss worldwide and pose a serious threat to aquatic life (Sustainable Development Goal 14)^1^. One of the best-documented, as well as most damaging, invasions recorded to date involves the Indo-Pacific lionfish (*Pterois* spp.), which are known to have negative impacts on the environment, economy, and human health in the Western Atlantic and Mediterranean Sea^2–6^. The establishment and expansion of lionfish populations across their non-native range have caused economic losses estimated at US$ 24 million per year^7^.

The rapid spread and successful establishment of lionfish in their non-native range is related to their broad dietary breadth, predation efficiency, large environmental niche, high fecundity, fast growth, resistance to parasites, and lack of natural predators in the invaded ecosystems^8^. Another possible component of this “ecological success equation” is that all Atlantic lionfish are likely to have a hybrid origin, which could have provided adaptive advantages for the founders of the invasive population^9,10^.

In the South Atlantic Ocean, two lionfish (both identified as *Pterois volitans*) were first detected in Arraial do Cabo (Eastern Brazil ecoregion *sensu* Spalding et al.^11^, Rio de Janeiro coast), one in 2014 and another in 2015^12,13^. Due to the distance of this region from the Caribbean (5500 km), it remained unclear whether these sightings represented a long-distance dispersal event or a secondary release of animals from the aquarium trade^12^. As no further lionfish specimens were detected anywhere in this Brazilian region during the subsequent 8 years, an aquarium release would appear to be the most likely explanation^13^.

However, the scenario has changed dramatically in recent years. Between 2020 and 2023 lionfish have spread throughout the SW Atlantic, with multiple records over an extensive area (~ 2800 km of coast) and depth range (1–110 m) including Great Amazon Reef System (GARS), the northeastern coast of Brazil (NEB), and the oceanic archipelago of Fernando de Noronha (FNA)^13–15^. Now fully fledged, this invasion has been expected for 10 years, ever since lionfish populations were established at the southernmost limit of the Caribbean Province, Trinidad and Tobago^13^. This delay was related to the process of overcoming the porous biogeographical barrier of the Amazon-Orinoco plume^16^. The first records of lionfish in the equatorial SW Atlantic were obtained in 2020 from the Amazon mesophotic reefs at depths of 70–100 m^13^. Since then, multiple records, involving approximately 350 individuals, have been reported from the equatorial SW Atlantic in 2020–2023^15^. Alarmingly, lionfish have recently reached the Rocas Reef complex, which is the only atoll in the South Atlantic Ocean. It seems highly likely that the recent sightings represent the presence of a consolidated population with larger and mature animals^14^. Furthermore, they may in fact represent the expansion phase of an invasive event initiated in 2020, on the Amazonian reefs. Herein, we hypothesise that adult lionfish have migrated from the Caribbean Sea, overcoming the porous Amazon-Orinoco biogeographical barrier by using the local mesophotic reefs as stepping stones, as previously suggested by Luiz et al.^16^.

Given this background, the present study applied genetic markers to describe the early stages of the lionfish bioinvasion of the equatorial SW Atlantic and the Brazilian coast, to determine the origin and diversity of the invading fish, and potential dispersal routes, as well as to aid in the management of this ongoing bioinvasion. Mitochondrial and nuclear DNA markers were sequenced in invasive lionfish specimens from the Brazilian coast and compared with *Pterois* spp. sequences available from the public NCBI and BOLD databases for identification purposes and to better elucidate the invasion and establishment of lionfish in the SW Atlantic.

## Results

**Figure 1:**
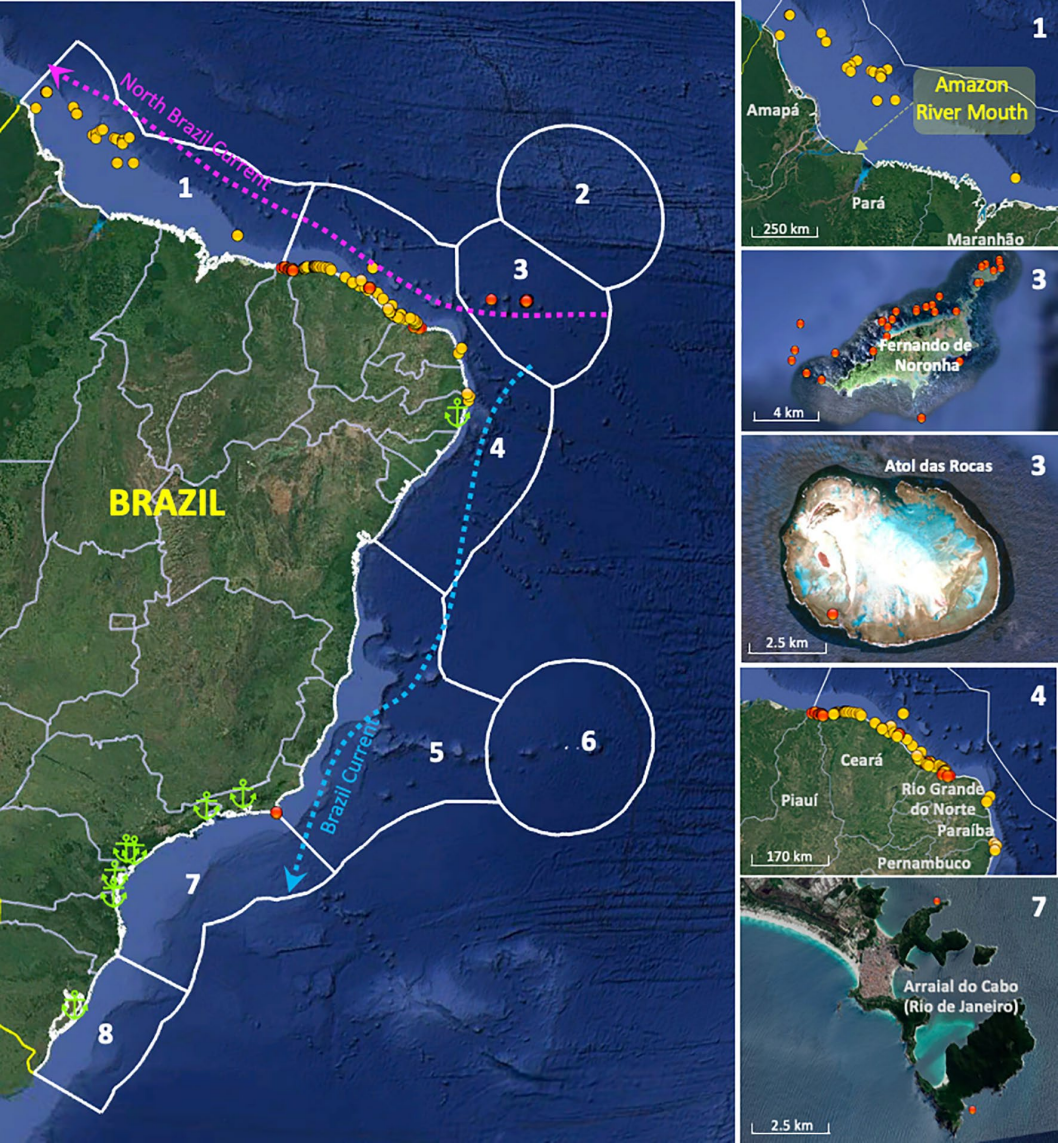
Ecoregions and main currents over the Brazilian Coast. Red and yellow dots indicate all recorded sightings and captures of lionfish; red dots indicate marine protected areas; samples that were analysed genetically are from ecoregions 1, 3 and 4. Green anchor icons indicate the location of the eight major Brazilian harbours. The images were retrieved from Google Earth. Map data ©2023 Google Image Landsat/Copernicus. Data SIO, NOAA, U.S. Navy, NGA, GEBCO.

**Figure 2:**
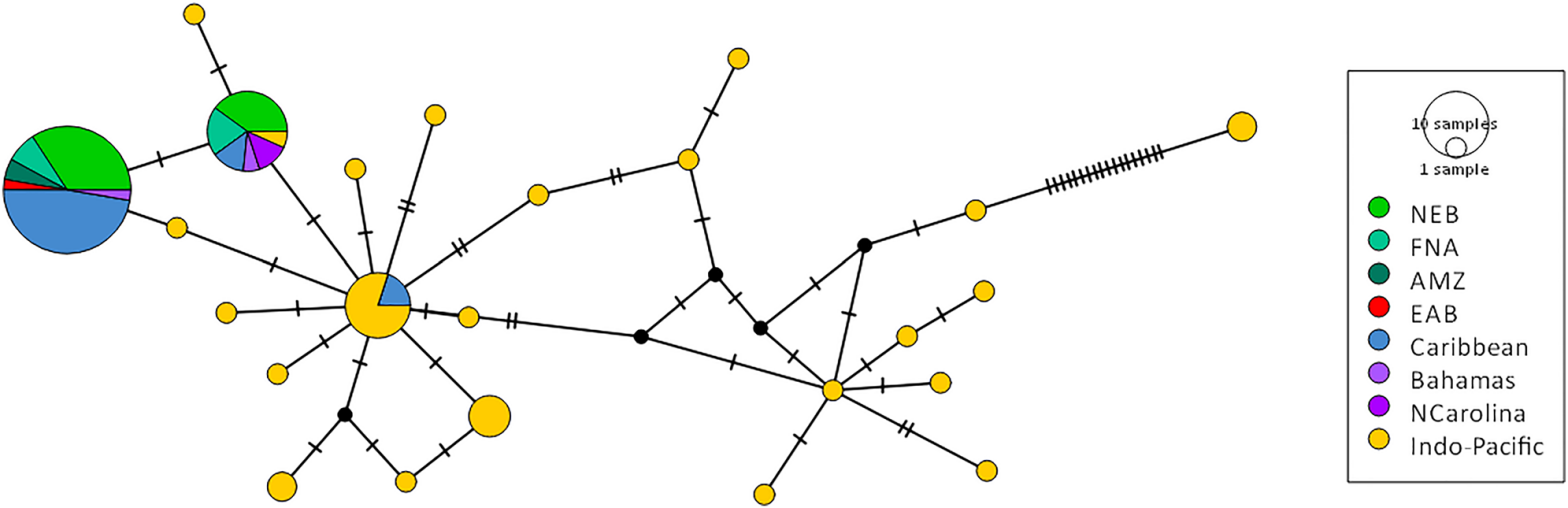
Median joining network for *P. volitans* COI sequences, including publicly available sequences (from BOLD, Wilcox et al.^10^, and others; see Supplementary Table 1). The network illustrates mostly haplotype diversity and relationships, since circle sizes are representative of sample size only for NEB, FNA and AMZ. *NEB* Northeast Brazil ecoregion, *FNA* Fernando de Noronha and Rocas Atoll ecoregion, *AMZ* Amazonia ecoregion, *EAB* Eastern Brazil ecoregion. Crossbars indicate single substitutions.

A total of 38 sequences of mitochondrial cytochrome c oxidase gene (COI) and 26 sequences of intron 1 of the S7 ribosomal protein gene (S7 RP1) of *Pterois* spp. were generated from samples obtained at various Brazilian locations, within three ecoregions (Fig. 1, Supplementary Table 1). These sequences indicate that all collected lionfish belong to what is generally accepted as *Pterois volitans*. COI partial sequences (577–655 bp; Supplementary Table 1) uncovered two haplotypes (Fig. 2). These haplotypes are the same as those recorded previously in the United States and the Caribbean, the most frequent of those being the same as that recorded previously in southeast Brazil, in 2014–2015^12^.

**Figure 3:**
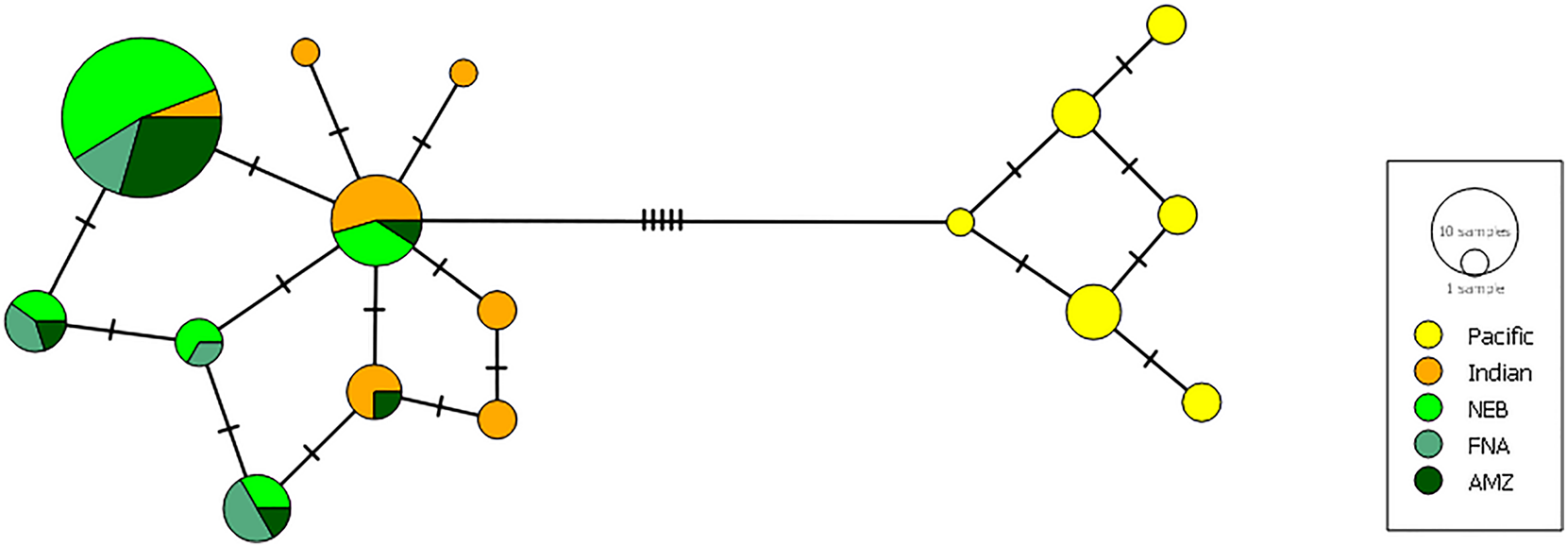
Median joining network for *P. volitans* S7 RP1 alleles, including only unambiguous allele sequences from Wilcox et al.^10^. The network illustrates mostly allele diversity and relationships, since circle sizes are representative of sample size only for NEB, FNA and AMZ. *NEB* Northeast Brazil ecoregion, *FNA* Fernando de Noronha and Rocas Atoll ecoregion, *AMZ* Amazonia ecoregion. Crossbars indicate single substitutions.

For S7 RP1, sequences of 503–728 bp (Supplementary Table 1) revealed six alleles, three of which are reported here for the first time (Fig. 3). Two of these newly found alleles were present in all three studied ecoregions, while the third one, only in NEB and FNA. The most frequent S7 RP1 allele found in Brazil (OP895002) corresponds to *P. miles* allele Ind07 (KT358620), which has been recorded in *P. volitans* as well^10^.

## Discussion

Our results provide the first molecular description of the early stages of lionfish invasion (2020–2022) in three ecoregions of the SW Atlantic (*sensu* Spalding et al.^11^), namely Amazonia, Northeastern Brazil and Fernando de Noronha and Rocas Atoll. Our novel results indicate that: (1) the species is *Pterois volitans* with a strong genetic affinity with the Caribbean population; and (2) despite the wide area (1800 km) and multiple shallow and mesophotic habitats sampled around Brazil, the same genetic signature was found in all specimens analysed, which indicates a single, rapid and successful invasion. Furthermore, previously unreported alleles found in the lionfish sampled in the study area could indicate population expansion, which would be consistent with the high invasive capacity of these fish, verified previously in the Western Atlantic and Mediterranean Sea.

A survey of genus *Pterois* over its native range described 19 COI haplotypes and 35 S7 RP1 alleles for *P. volitans*^10^. While the invasion of the Caribbean Sea involved both *Pterois volitans* and *Pterois miles*, mitochondrial data indicated that they are predominantly (> 90%) *P. volitans*^17^, which was later identified as a hybrid^10^. Recently, an analysis of the specimens from the Caribbean Sea, Florida, and northern USA showed alternative invasion scenarios that suggest separated secondary introductions^18^. These findings should be further investigated to better understand population boundaries and dispersal patterns, which are essential parameters for the effective control and management of future invasions. Nevertheless, mitochondrial COI data presented here indicate that all captured lionfish in the equatorial SW Atlantic belong to what is generally considered *Pterois volitans.* Furthermore, it supports the hypothesis of a single route of invasion from the Caribbean, because the only two haplotypes found for this gene are the same as those previously reported for the Bahamas and the Caribbean^10^.

The number of alleles and haplotypes are likely to increase in rapidly expanding populations, especially for quasi-neutral regions of the genome, such as introns. A large number of alleles have been reported for S7 RP1 over *P. volitans* native range, while six alleles were found in a North Carolina sample, two of them unique to the invaded location^10^. However, it cannot be said that *P. volitans* source populations have been fully described for S7 RP1. The data presented here describes an initial sample of the invading fish. Nevertheless, it revealed three previously unrecorded alleles. In this scenario, our data seems likely to underrepresent S7 RP1 variability in the Brazilian ecoregions. A high variability in S7 RP1 would be consistent with the rapid expanding lionfish invasion observed now^14,15^. It seems clear that in order to understand the demography of the invading lionfish using S7 RP1, a deeper understanding of its mutation rates and a wider and continued genetic survey is necessary. Finally it is important to highlight that we found the predominance of an S7 RP1 allele previously attributed to *P. miles* from the Indian Ocean, which seems to support a hybrid origin of the SW Atlantic lionfish, as proposed by Wilcox et al.^10^.

Four hypotheses can be proposed to account for the recent occurrences of lionfish in the equatorial SW Atlantic (Brazil): (1) passive transfer in ballast water discharged by ships in ports, (2) aquarium release, (3) larvae dispersal, and (4) adult movement from the Caribbean, a neighbouring region interconnected to the equatorial SW Atlantic. The first two hypotheses seem unlikely. There are no records of lionfish presence associated with major Brazilian ports, such as Mucuripe harbour, on the Ceará coast^13,14^. Similarly, there is no evidence of recent transactions involving adult lionfish in the ornamental fish trade in any of the regions affected, and they would be especially unlikely in Fernando de Noronha (oceanic region). As a matter of fact, after the first lionfish was found in Brazilian waters^12^, it raised concerns on the risk of bioinvasion related to possible aquarium releases, and the Brazilian environmental authority banned the import of five *Pterois* species. Before that, starting in 2008, all imports had to be registered^15^. On the other hand, larval dispersion from the Caribbean would require countercurrent movement over thousands of kilometres and a successful crossing of the Amazon plume, which flows northward into the Caribbean^16^. Therefore, the most parsimonious explanation would appear to be that adult lionfish have migrated from the Caribbean Sea, possibly overcoming the porous Amazon-Orinoco biogeographical barrier using the local mesophotic reefs as stepping stones, as previously suggested by Luiz et al.^16^. This hypothesis is also strongly supported by the spatio-temporal trends recently observed for the lionfish invasion in Brazilian waters and by the genetic data presented here. In the past three years (2020–2023) 352 animals, including juveniles, adults, and egg-bearing females, were reported on 2766 km of Brazilian coast, which strongly indicates a fast and extensive new stage of the SW Atlantic invasion^14,15^. While some diversification is suggested by the nuclear S7 RP1, the two COI haplotypes observed in our samples have been previously reported in the United States and the Caribbean, seemingly connecting the population studied here to that present in the Caribbean. Population bottlenecks due to founder effect are expected in the early stages of an invasion. Herein, no genetic evidence of bottleneck was found, and the observed diversity does not seem to hinder the invasive capability of these first specimens recorded in Brazilian waters.

Indeed, environmental conditions and historical records indicate that the ongoing lionfish invasion likely initiated with adult individuals of *P. volitans* that crossed the biogeographic barrier of the Amazon River and reached the equatorial SW Atlantic through the reefs located underneath the estuarine river plume in a stepping-stone movement. The turbid and brackish waters of the Amazon-Orinoco plume extend some 2300 km along the coast of northern South America and represent an effective barrier for taxa with reduced dispersal capacity. However, the water that lies under the plume is more saline, which, along with the large Amazon reef system below, may facilitate the exchange of marine species in these subsurface waters^19^. Moreover, the North Brazil Current, flowing from the North of Brazil towards the Caribbean, probably plays a major role in this biogeographical boundary, driving the flow of larvae and other planktonic organisms with reduced mobility northwestward, and hindering their movement in the opposite direction. Nevertheless, pelagic taxa with active swimming capacity may be able to cross it^19^, as our genetic data suggest.

The function of mesophotic reefs in the Amazon and northeastern coast as stepping stones to migration in both directions of the Caribbean and Brazilian Province has been predicted earlier^20–22^, including for the lionfish invasion^16^. The main direction of coastal currents would not prevent the movement of adult individuals from one habitat to the next in a stepping-stone pattern^13,14,16^. This hypothesis was first proposed by Luiz et al.^13,16^ and Soares et al.^14^ and is supported by the results of genetic analyses herein. The expansion pattern suggested here seems similar to what is happening with the invading *Pterois miles* in the Mediterranean^23^, where lionfish followed a typical Lessepsian immigrant pattern starting in the Levantine Sea then gradually spreading into the eastern Mediterranean in a stepping stone pattern^23^. This pattern reflects the adaptive capacity of these invasive fish and the risks of their expansion around the world.

The temporal and spatial distribution of the recent records, with lionfish first being sighted on Amazon mesophotic reefs in 2020, gradually spreading into the oceanic archipelago^13^, and then to the Brazilian northeastern coast^14^, is also consistent with a stepping-stone invasion of the equatorial SW Atlantic through the Amazon reef corridor underneath the estuarine river plume and the semi-continuous mesophotic reef system along the Brazilian equatorial coast^24^. Moreover, larger animals (20–40 cm) are found off the Amazon coast in comparison with the populations from northeastern Brazil and Fernando de Noronha archipelago (20–30 cm^15^). This evidence suggests an earlier arrival and corresponding older lionfish population in the Amazon coast, which supports the adult dispersal hypothesis indicated by our genetic results.

We found mostly the same COI haplotypes and S7 RP1 alleles in samples from three ecoregions (Amazonia, Northeastern Brazil and Fernando de Noronha and Rocas Atoll) despite distances of almost 1800 km between our genetic samples. This could be explained by currents (such as the South Equatorial current), as well as reefs and seamounts that link these environments in the SW Atlantic. The Brazilian equatorial margin (Ceará plateau) has extensive seamounts and reef habitats located 20–50 m deep on the continental shelf between the GARS and the Eastern Brazilian Reef System^24^. This is the largest cluster of seamounts in the equatorial SW Atlantic and the closest plateau to the continent^25^, which forms part of the volcanic mountain chain of the Fernando de Noronha Archipelago, providing extensive shallow and mesophotic reef habitats for lionfish adult dispersal. Therefore, lionfish have likely used the mesophotic reefs and seamounts from the equatorial SW Atlantic (Ceará and Piauí)^14,26^ as stepping-stones to arrive in Fernando de Noronha Archipelago. It is also likely that they are using artificial reefs (e.g., oil and gas platforms, and shipwrecks) as additional habitats, which help them recruit and establish populations in new environments along the equatorial SW Atlantic coast, as already observed for the invasive coral, *Tubastraea* spp*.*^27^.

Starting in 2020, new lionfish sightings and captures in the Brazilian waters accumulated rapidly, with more than 350 individuals reported along almost 3000 km of coastline^15^; we report here that a few months ago, lionfish was seen for the first time in the remote Rocas Atoll (Fig. 1) and in Costa dos Corais, the largest nearshore Brazilian marine protected area. Lionfish is now present in 14 protected areas and eight Brazilian states and the invasion is rapidly progressing southward towards Eastern Brazil, which harbours important marine biodiversity hotspots, such as Abrolhos Bank, and Trindade and Martin Vaz Islands^15^. We presented here the first approach to the genetics of this stage of the SW Atlantic invasion, using basic genetic tools that should be further explored in subsequent studies. Invasion genomics is an emerging field, where genomic tools, such as monitoring of sensitive areas through environmental DNA, can give insights into spread patterns of non-indigenous species in their introduced range, thus improving management effectiveness and control plans for ongoing or future invasions. Moreover, those tools can be used to investigate other genetic parameters, such as effective population size and inbreeding coefficient of invading populations. Genomics and bioinformatics will provide valuable knowledge about the demography and diversification of invasive species, especially if supported by continued surveying. However, control actions in high-priority Brazilian sites, with the support of fishers and divers, will be fundamental to mitigate the impacts of the lionfish invasion^14^.

## Methods

### Sampling

Lionfish samples were obtained along the equatorial coast of the SW Atlantic at different locations and habitats, including the Great Amazon Reef System—GARS (Brazilian state of Pará), the semiarid coast of northeastern Brazil (Piauí, Ceará and, Rio Grande do Norte states), and the Fernando de Noronha Archipelago, covering three marine ecoregions (*sensu* Spalding et al.^11^; Fig. 1). A total of 32 specimens were obtained from Pará state (Amazonia ecoregion), 21 from Ceará, Piauí, and Rio Grande do Norte states (Northeastern Brazil ecoregion), and 14 from the Fernando de Noronha Archipelago (Fernando de Noronha and Rocas Atoll ecoregion).

The sampling covered an extensive area of about 1800 km between 2020 and 2022 and was based on field surveys together with fishers and divers (Fig. 1, Supplementary Table 1). All specimens used in this study were morphologically identified as red lionfish, *Pterois volitans*. The samples of muscle tissue were preserved in 95% ethanol and transported to the laboratory for DNA extraction. All samples were stored at − 20 °C until laboratory analyses.

### DNA extractions, PCR amplification, and sequencing

Genomic DNA was extracted using Phenol–Chloroform Extraction, followed by genomic DNA precipitation and resuspension in TE. The final concentration and purity of DNA samples were determined using a Nanodrop spectrophotometer (Thermo Scientific, USA). Two loci were used as genetic markers to distinguish *Pterois* specimens. One marker was the mitochondrial cytochrome c oxidase, subunit I gene (COI) that was amplified by PCR using the primers FishF2 and FishR1 described by Ward et al.^28^. The second marker was intron 1 of the nuclear S7 ribosomal protein (S7 RP1), which was amplified using RP1F and RP1R primers described by Chow and Hazama^29^. Each PCR contained 10 ng of DNA, 1 × Buffer, 1.5 mM of MgCl_2_, 0.2 mM of dNTP, 0.2 mM of each primer, 1 U of Taq DNA polymerase, and ultrapure water to complete the final volume of 25 µL. Following purification, amplicons were sequenced in both directions using the fluorescent dideoxynucleotides Sanger method at a third-party partner. All sequences were deposited in GenBank under the accession numbers OP882709–OP882735 and OQ750554–OQ750564 (COI sequences); and OP894998–OP895014 and OQ800937–OQ800945 (S7 RP1 sequences) (NCBI Bioproject PRJNA954392).

### Data analyses

Sequencing reads were checked manually, edited, and aligned in MEGA11^30^. The haplotypes and alleles identified in each sample were used to produce reticulate trees in PopART^31^ using the Median-joining method. *Pterois* spp. publicly available sequences from NCBI (https://www.ncbi.nlm.nih.gov/) and BOLD databases (https://www.boldsystems.org/) were included in this analysis (see Supplementary Table 2 for further information).

### Ethical statement

The authors declare no conflict with ARRIVE guidelines since the samples did not originate from experimentation on live animals. The species in question is an invasive species, which is neither endangered nor threatened.

### Supplementary Information

Supplementary Tables.

## Data Availability

All sequences were deposited in GenBank under the Accession Numbers OP882709–OP882735 and OQ750554–OQ750564 (COI sequences); and OP894998–OP895014 and OQ800937–OQ800945 (S7 RP1 sequences) (NCBI Bioproject PRJNA954392). Supplementary Material is available online.
