# Supplementary material for: Genetic diversity patterns of lionfish in the Southwestern Atlantic Ocean reveal a rapidly expanding stepping-stone bioinvasion process
Source: Sci Rep. 2023 Aug 18;13:13469. doi: 10.1038/s41598-023-40407-y (PMC10439126; doi:10.1038/s41598-023-40407-y)
Supplement: Supplementary file 1 — Supplementary Tables. [file 41598_2023_40407_MOESM1_ESM.docx]

**Supplementary material**

Genetic diversity patterns of lionfish in the Southwestern Atlantic Ocean reveal a rapidly expanding stepping-stone bioinvasion process

# Rodrigo Maggioni ^1*^, Rafael S. Rocha ^1^, Jhonatas T. Viana^1^, Tommaso Giarrizzo ^1, 2^, Emanuelle F. Rabelo ^3^, Carlos E. L. Ferreira ^4^, Claudio L. S. Sampaio ^5^, Pedro H. C. Pereira ^6^, Luiz A. Rocha ^7^, Tallita C. L. Tavares ^1^, Marcelo O. Soares ^1, 8, 9 *^

1Instituto de Ciências do Mar (LABOMAR), Universidade Federal do Ceará (UFC), Avenida da Abolição, 3207, Fortaleza, Brazil

2Núcleo de Ecologia Aquática e Pesca da Amazônia (NEAP), Universidade Federal do Pará (UFPA), Belém, PA, Brazil

^3^ Universidade Federal Rural do Semiárido (UFERSA), Mossoró, Brazil

^4^ Laboratório de Ecologia e Conservação de Ambientes Recifais (LECAR), Departamento de Biologia Marinha, Universidade Federal Fluminense (UFF), Niterói, RJ, Brazil

^5^ Universidade Federal de Alagoas (UFAL), Unidade Penedo, Alagoas, Brazil

^6^ Projeto Conservação Recifal (PCR), Recife, Brazil

^7^ California Academy of Sciences, San Francisco, United States of America

^8^ Reef Systems Group, Leibniz Center for Tropical Marine Research (ZMT), Bremen, Germany

^9^ Center for Marine and Environmental Studies (CMES), University of the Virgin Islands (UVI), St. Thomas, United States Virgin Islands

*[corresponding authors: maggioni@ufc.br; marcelo.soares@leibniz-zmt.de](mailto:corresponding%20authors:%20maggioni@ufc.br;%20marcelo.soares@leibniz-zmt.de)

**Supplementary Table 1**. Sample sites and sequence information. NEB, Northeast Brazil ecoregion; FNA, Fernando de Noronha and Rocas Atoll ecoregion; AMZ, Amazonia ecoregion; EAB, Eastern Brazil ecoregion.

| **ID** | **Location** | **Accession**  **(COI/S7RP1)** | **COI (bp)** | **S7RP1 (bp)** | **Geog Coord** | **Region** |
| --- | --- | --- | --- | --- | --- | --- |
| 01 | Icapuí | OP882717 / n.a. | 655 | - | 4°40'12.0"S 37°25'12.0"W | NEB |
| 02 | Icapuí | OP882718 / OP894998 | 655 | 610 | 4°40'48.0"S 37°23'24.0"W | NEB |
| 03 | Icapuí | OP882719 / n.a. | 655 | - | 4°40'48.0"S 37°22'48.0"W | NEB |
| 04 | Icapuí | OP882720 / n.a. | 624 | ? | 4°36'00.0"S 37°14'24.0"W | NEB |
| 05 | Parnaíba | OP882721 / n.a. | 655 | ? | 2°51'36.0"S 41°20'24.0"W | NEB |
| 06 | Parnaíba | OP882722 / OP894999 | 655 | 656 | 2°51'36.0"S 41°38'24.0"W | NEB |
| 07 | Preá | OP882723 / OP895000 | 655 | 614 | 2°48'36.0"S 40°25'12.0"W | NEB |
| 08 | Preá | OP882724 / OP895001 | 655 | 679 | 2°48'36.0"S 40°25'12.0"W | NEB |
| 09 | Camocim | OP882725 / OP895002 | 655 | 662 | 2°51'00.0"S 40°46'48.0"W | NEB |
| 10 | Camocim | OP882726 / OP895003 | 655 | 680 | 2°51'00.0"S 40°46'48.0"W | NEB |
| 11 | Camocim | OP882727 / OP895004 | 655 | 728 | 2°51'00.0"S 40°46'48.0"W | NEB |
| 12 | Camocim | OP882728 / OP895005 | 655 | 642 | 2°51'00.0"S 40°46'48.0"W | NEB |
| 13 | Arpoeiras | OP882729 / n.a. | 655 | - | 2°48' 51.1'' S 40°07' 03.4'' W | NEB |
| 14 | Arpoeiras | OP882730 / OP895006 | 655 | 630 | 2°48' 51.1'' S 40°07' 03.4'' W | NEB |
| 15 | Lagoa da Volta | OP882731 / OP895007 | 655 | 682 | 2°48.075' S 40°14.988' W | NEB |
| 16 | Lagoa da Volta | OP882732 / OP895008 | 655 | 596 | 2°48.075' S 40°14.988' W | NEB |
| 17 | Ilha dos Coqueiros | OP882733 / OP895009 | 655 | 631 | 2°50' S  40°01' W | NEB |
| 18 | Ilha dos Coqueiros | OP882734 / OP895010 | 655 | 557 | 2°50' S  40°01' W | NEB |
| 19 | Itarema | OP882735 / n.a. | 655 | ? | 2°51'00.0"S 39°52'48.0"W | NEB |
| 20 | Camocim | n.a. / n.a. | ? | - | 2°52'12.0"S 40°48'36.0"W | NEB |
| 21 | Camocim | n.a. / n.a. | ? | - | 2°52'12.0"S 40°48'36.0"W | NEB |
| 22 | Fernando de Noronha | OP882709 / n.a. | 655 | ? | 3°49'59.34"S 32°25'15.02"W | FNA |
| 23 | Fernando de Noronha | OP882710 / OP895011 | 577 | 619 | 3°49'59.34"S 32°25'15.02"W | FNA |
| 24 | Fernando de Noronha | OP882711 / n.a. | 604 | ? | 3°48'28.92"S 32°23'23.06"W | FNA |
| 25 | Fernando de Noronha | OP882712 / OP895012 | 626 | 633 | 3°49'59.34"S 32°25'15.02"W | FNA |
| 26 | Fernando de Noronha | OP882713 / OP895013 | 655 | 644 | 3°50'44.12"S 32°26'29.08"W | FNA |
| 27 | Fernando de Noronha | OP882714 / n.a. | 593 | ? | 3°50'09.39"S 32°25'34.32"W | FNA |
| 28 | Fernando de Noronha | n.a. / OP895014 | ? | 636 | 3°49'59.34"S 32°25'15.02"W | FNA |
| 29 | Pará | OP882715 / n.a. | 655 | ? | 1°23'42.0"N 47°22'15.6"W | AMZ |
| 30 | Fernando de Noronha | n.a. / n.a. | - | - | 3°50'34.92"S 32°26'42.10"W | FNA |
| 31 | Fernando de Noronha | n.a. / n.a. | - | - | 3°48'26.89"S 32°23'23.71"W | FNA |
| 32 | Fernando de Noronha | n.a. / n.a. | - | - | 3°48'26.89"S 32°23'23.71"W | FNA |
| 33 | Fernando de Noronha | n.a. / n.a. | - | - | 3°49'01.75"S 32°23'25.45"W | FNA |
| 34 | Fernando de Noronha | n.a. / n.a. | - | - | 3°50'34.92"S 32°26'42.10"W | FNA |
| 35 | Fernando de Noronha | n.a. / n.a. | - | - | 3°48'28.92"S 32°23'23.06"W | FNA |
| 36 | Pará | OP882716 / n.a. | 618 | ? | 4°41'24.0"N 50°25'33.6"W | AMZ |
| 37 | Fernando de Noronha | n.a. / n.a. | - | - | 3°50'03.95"S 32°24'13.34"W | FNA |
| A03 | Pará | OQ750554 / OQ800937 | 655 | 511 | 4°41'24.0"N 50°25'33.6"W | AMZ |
| A04 | Pará | OQ750555 / OQ800938 | 655 | 544 | 4°41'24.0"N 50°25'33.6"W | AMZ |
| A08 | Pará | OQ750556 / OQ800939 | 655 | 578 | 4°41'24.0"N 50°25'33.6"W | AMZ |
| A13 | Pará | OQ750557 / OQ800940 | 655 | 523 | 4°41'24.0"N 50°25'33.6"W | AMZ |
| A22 | Pará | OQ750558 / OQ800941 | 655 | 561 | 4°41'24.0"N 50°25'33.6"W | AMZ |
| A23 | Pará | OQ750559 / n.a. | 655 | ? | 4°41'24.0"N 50°25'33.6"W | AMZ |
| A25 | Pará | OQ750560 / OQ800942 | 655 | 503 | 4°41'24.0"N 50°25'33.6"W | AMZ |
| A27 | Pará | OQ750561 / OQ800943 | 655 | 601 | 4°41'24.0"N 50°25'33.6"W | AMZ |
| A32 | Pará | OQ750562 / n.a. | 655 | ? | 4°41'24.0"N 50°25'33.6"W | AMZ |
| A22 | Fernando de Noronha | OQ750563 / OQ800944 | 655 | 585 | 4°41'24.0"N 50°25'33.6"W | FNA |
| A02 | Rio Grande do Norte | OQ750564 / OQ800945 | 655 | 566 | 4°48'57.0"S 36°44'97.0"W | NEB |

“?” represents PCR amplified, failed sequencing; “-” represents no PCR amplification; n.a., not available

**Supplementary Table 2.** List of publicly available sequences used in the present work.

| **Gene** | **Accession** | **Database** | **Location** |
| --- | --- | --- | --- |
| COI | BAHB277-15 | BOLD | Bahamas |
| COI | BAHB278-15 | BOLD | Bahamas |
| COI | BCOLL183-06 | BOLD | North Carolina |
| COI | FNZ215-06 | BOLD | Indo-Pacific |
| COI | FOAI061-08 | BOLD | Indo-Pacific |
| COI | FOAJ750-09 | BOLD | Indo-Pacific |
| COI | FOAM235-10 | BOLD | Indo-Pacific |
| COI | FOAO1310-18 | BOLD | Indo-Pacific |
| COI | FOAO1947-19 | BOLD | Indo-Pacific |
| COI | FUT300-18 | BOLD | Indo-Pacific |
| COI | GBGCA12790-15 | BOLD | Eastern Brazil |
| COI | GBMNB9381-20 | BOLD | Indo-Pacific |
| COI | GBMTG4364-16 | BOLD | North Carolina |
| COI | ISABZ189-19 | BOLD | Caribbean |
| COI | KT358509.1 | GenBank | Indo-Pacific |
| COI | KT358517.1 | GenBank | Indo-Pacific |
| COI | KT358518.1 | GenBank | Indo-Pacific |
| COI | KT358520.1 | GenBank | Indo-Pacific |
| COI | KT358521.1 | GenBank | Indo-Pacific |
| COI | KT358525.1 | GenBank | Indo-Pacific |
| COI | KT358527.1 | GenBank | Indo-Pacific |
| COI | KT358528.1 | GenBank | Indo-Pacific |
| COI | KT358529.1 | GenBank | Indo-Pacific |
| COI | KT358530.1 | GenBank | Indo-Pacific |
| COI | KT358531.1 | GenBank | Indo-Pacific |
| COI | KT358532.1 | GenBank | Indo-Pacific |
| COI | KT358533.1 | GenBank | Indo-Pacific |
| COI | KT358534.1 | GenBank | Indo-Pacific |
| COI | KT358535.1 | GenBank | Indo-Pacific |
| COI | KT358536.1 | GenBank | Indo-Pacific |
| COI | KT358537.1 | GenBank | Indo-Pacific |
| COI | KT358538.1 | GenBank | Indo-Pacific |
| COI | KT358539.1 | GenBank | Indo-Pacific |
| COI | KT358540.1 | GenBank | Indo-Pacific |
| COI | KT358541.1 | GenBank | Indo-Pacific |
| COI | LIFS845-08 | BOLD | Indo-Pacific |
| COI | MFLS173-10 | BOLD | Caribbean |
| COI | MXII616-10 | BOLD | Caribbean |
| COI | MXIV209-10 | BOLD | Caribbean |
| COI | MXIV226-10 | BOLD | Caribbean |
| COI | MXIV230-10 | BOLD | Caribbean |
| COI | MXIV242-10 | BOLD | Caribbean |
| COI | MXIV243-10 | BOLD | Caribbean |
| COI | MXIV244-10 | BOLD | Caribbean |
| COI | MXIV250-10 | BOLD | Caribbean |
| COI | MXIV254-10 | BOLD | Caribbean |
| COI | MXIV255-10 | BOLD | Caribbean |
| COI | MXIV487-10 | BOLD | Caribbean |
| COI | MXIV490-10 | BOLD | Caribbean |
| COI | MXIV492-10 | BOLD | Caribbean |
| COI | MXIV498-10 | BOLD | Caribbean |
| COI | MXIV501-10 | BOLD | Caribbean |
| COI | MXIV569-11 | BOLD | Caribbean |
| COI | MXV122-11 | BOLD | Caribbean |
| COI | MXV227-11 | BOLD | Caribbean |
| COI | MXV371-11 | BOLD | Caribbean |
| COI | TZAIB466-06 | BOLD | Indo-Pacific |
| COI | TZAIB691-06 | BOLD | Indo-Pacific |
| COI | TZAIB692-06 | BOLD | Indo-Pacific |
| COI | TZAIB810-07 | BOLD | Indo-Pacific |
| COI | TZAIC905-06 | BOLD | Indo-Pacific |
| COI | UKFBJ1201-08 | BOLD | North Carolina |
| S7RP1 | KT358587.1 | GenBank | Pacific |
| S7RP1 | KT358589.1 | GenBank | Pacific |
| S7RP1 | KT358590.1 | GenBank | Pacific |
| S7RP1 | KT358591.1 | GenBank | Pacific |
| S7RP1 | KT358593.1 | GenBank | Pacific |
| S7RP1 | KT358594.1 | GenBank | Pacific |
| S7RP1 | KT358596.1 | GenBank | Pacific |
| S7RP1 | KT358599.1 | GenBank | Pacific |
| S7RP1 | KT358606.1 | GenBank | Pacific |
| S7RP1 | KT358616.1 | GenBank | Indian |
| S7RP1 | KT358617.1 | GenBank | Indian |
| S7RP1 | KT358618.1 | GenBank | Indian |
| S7RP1 | KT358620.1 | GenBank | Indian |
| S7RP1 | KT358622.1 | GenBank | Indian |
| S7RP1 | KT358623.1 | GenBank | Indian |
| S7RP1 | KT358624.1 | GenBank | Indian |
| S7RP1 | KT358625.1 | GenBank | Indian |
| S7RP1 | KT358626.1 | GenBank | Indian |
| S7RP1 | KT358631.1 | GenBank | Indian |
| S7RP1 | KT358635.1 | GenBank | Indian |
